# HABIT efficacy and sustainability trial, a multi-center randomized controlled trial to improve hydroxyurea adherence in youth with sickle cell disease: a study protocol

**DOI:** 10.1186/s12887-019-1746-6

**Published:** 2019-10-15

**Authors:** Arlene Smaldone, Deepa Manwani, Banu Aygun, Kim Smith-Whitley, Haomiao Jia, Jean-Marie Bruzzese, Sally Findley, Joshua Massei, Nancy S. Green

**Affiliations:** 1 Columbia University School of Nursing, New York, NY USA; 2 Columbia University College of Dental Medicine, New York, NY USA; 3 Albert Einstein College of Medicine, Bronx, NY USA; 4 Zucker School of Medicine at Hofstra/Northwell, New Hyde Park, New York, USA; 5 Perelman School of Medicine, Philadelphia, PA USA; 6 Mailman School of Public Health, New York, NY USA; 7 Vagelos College of Physicians and Surgeons, Columbia University, New York, NY USA

**Keywords:** Sickle cell disease, Hydroxyurea, Community health workers, Mobile health, Randomized controlled trial, Adherence, Health related quality of life

## Abstract

**Background:**

Hydroxyurea (HU) is recommended as standard practice for youth with sickle cell disease (SCD). Yet, despite its efficacy, HU adherence in adolescents and young adults is often poor. Poor medication adherence increases disease burden, healthcare cost and widens health disparities. Adolescence is a critical time to improve adherence through improved chronic disease self-management. This study aims to test the efficacy of an intervention delivered to youth/parent dyads by community health workers (CHWs), augmented by tailored text messages on HU adherence (primary outcome). Secondary outcomes are intervention sustainability, youth health-related quality of life, self-management responsibility concordance, acute hospital use and self-reported disease symptoms.

**Methods:**

Hydroxyurea Adherence for Personal Best in Sickle Cell Disease, “HABIT,” is a 12 month multi-center randomized controlled trial. One hundred four youth, 10 to 18 years of age prescribed HU who meet eligibility criteria, enrolled with their parent as dyads, will be randomized 1:1 to either the HABIT intervention or to usual clinical care plus education handouts. All subjects will complete clinic visits at months 0, 2, 4, 6 (efficacy component), 9 and 12 (sustainability component) for assessment of HbF biomarker, other hematologic parameters, and to complete questionnaires. In addition, dyads assigned to the HABIT intervention will work with CHWs to identify a daily habit (e.g., brushing teeth) on which to build a HU adherence habit. Tailored daily text message reminders to support the habit will be developed by the dyad in collaboration with the CHWs and sent to parent and youth. At the 6 month visit, the intervention will end and the sustainability portion of the trial will begin. All data analyses will be based on intention to treat with all randomized subjects included in the analyses.

**Discussion:**

Prior retrospective studies demonstrate that a majority of adolescents are poorly adherent to HU. If efficacious, the HABIT intervention has the potential to improve the lives of youth with SCD.

**Trial registration:**

Clinicaltrials.gov NCT03462511. Registered March 6, 2018, last updated July 26, 2019.

## Background

Sickle cell disease (SCD) affects approximately 100,000 Americans of African descent including Caribbean Latinos and other underserved ethnicities [1, 2]. An inherited blood disease, SCD is characterized by fatigue, pain, organ damage, reduced health related quality of life (HRQOL), high health care costs and shortened lifespan [3–6]. Hydroxyurea (HU) is recommended as standard practice for youth with SCD [7]. HU markedly reduces symptoms, morbidity and mortality, improves QOL, decreases health cost, and may protect against cumulative disease burden [7, 8]. HU induces a dose-dependent increase in fetal hemoglobin (HbF), an effect that is largely responsible for its impact [9, 10]. Despite its benefits, HU adherence in adolescents and young adults is often poor [11–16].

Barriers to adherence, especially among underserved populations, include cultural misalignment with medical staff [17, 18], incomplete knowledge of drug benefit, concerns about toxicity [19–21], and logistic impediments to timely prescription refill [22]. Poor adherence is linked to inadequate integration of adherence into a daily medication habit [23]. Barriers to medication adherence are common in youth with chronic illness [15, 24–28] and a source of racial/ethnic disparities in underserved communities [29]. Poor medication adherence increases disease burden, healthcare cost and widens health disparities [30, 31].

Treatment adherence measures have included health care utilization, treatment success rates, school/work missed, bioassays and symptom improvement [32]. For SCD, poor HU adherence has been documented by pharmacy prescription refill databases [11, 33] and by reduction from peak HbF levels [14, 16], a sensitive biomarker for dose-dependent HU use [34]. To date, studies to improve HU adherence have been limited and have primarily relied on proximal outcomes [35–38] rather than broader patient-reported outcome measures and have not employed strategies to sustain longer-term self-management behaviors that are sensitive to socio-cultural patient and family needs.

Self-management is a set of behaviors that people living with chronic health conditions must incorporate into their lifestyle to optimize their health [39]. The roles of youth and parents in self-management change throughout childhood and adolescence with self-management responsibility transitioning from parent to youth. Chronic disease management often deteriorates during adolescence [26] when youth assume greater self-management responsibility [15, 20, 40–42]. Shared responsibility in youth-parent partnerships for self-management supports adolescents by a gradual transition to self-management [43–46]. Adolescence is a critical time to improve adherence. Ideally, during adolescence transfer of developmentally appropriate self-management responsibility occurs gradually with parents remaining involved in a supportive role [47]. However, this is often not the case. Adherence barriers as reported by adolescents with chronic health conditions were synthesized and reported in a systematic review of 28 studies [48]. Across studies of youth with a variety of chronic conditions, poor adherence was associated with adolescent-parent conflict; conflict stemmed from either parental difficulty with delegation of self-management responsibility or adolescent perception of lack of parental support. Maintaining self-management communication between parents and youth is key. Identifying successful ways to improve HU adherence through developmentally appropriate self-management has the potential to improve the health of youth with SCD.

While long-term pediatric clinical HU trials have demonstrated the stability of HbF levels over time [49–51], a uniform standard biomarker to assess HU adherence is lacking [10, 12, 14, 52]. In our earlier retrospective cohort study and in our Hydroxyurea Adherence for Personal Best in Sickle Cell Disease (HABIT) feasibility trial [53, 54], we validated the highest historical HU-induced HbF as an innovative biomarker for an individualized Personal Best self-management goal [16]. Personal Best HbF serves as a customized minimum target for adherence and can augment other blood cell measures that are typically used to assess adherence such as red blood cell volume and white blood cell count [50, 55]. During the HABIT feasibility trial, community health workers (CHWs) coached youth/parent dyads regarding their progress toward reaching their historical Personal Best HbF and found it to be a useful and acceptable way to communicate adherence progress.

Culturally aligned CHWs are an accepted mode of community-based support for improving health in underserved communities [56, 57] and bridge gaps between underserved patients and clinical staff. A body of literature supports the success of CHWs in working with vulnerable adults with chronic illness [58–60]. Research examining use of CHWs in children with chronic illness is more limited [61], particularly for youth with sickle cell disease [62]. In the HABIT feasibility trial, CHWs established trust with youth and parents and worked collaboratively with them to address barriers and improve HU adherence [54].

Texting health messages can be an effective way to send reminders from professionals to parents [63] and youth [64, 65] and is well accepted in our community [66]. Systematic reviews and meta-analyses of interventions to improve adherence among youth affected by chronic illness [67, 68] demonstrate the added value of multi-component interventions. The proposed study employs a two-component intervention to improve HU adherence: CHW support augmented by tailored text messages delivered to parent youth dyads. During the HABIT feasibility study, CHWs worked with HABIT dyads to design automated, cue-based text messages. As part of the proposed HABIT efficacy trial, an additional weekly text message will be sent to parents and youth to monitor HU use and adherence behavior. Dyad responses to the weekly text will inform the need for additional CHW support and identify dyads having problems with establishing a HU adherence habit. To our knowledge, an intervention integrating CHW support with text messaging has not been previously studied.

Building upon a successful trial to assess the feasibility and acceptability of the HABIT intervention [53, 54, 69], this paper describes the study protocol for a multi-center randomized controlled trial to test the efficacy and sustainability of HABIT, a CHW intervention augmented by tailored text messages, to improve HU adherence in youth age 10-18 years with SCD. Given the high prevalence of poor HU adherence among youth, the HABIT intervention could improve the lives of youth with SCD.

## Methods

### Aims and study hypotheses

This 12 month multi-center randomized controlled trial (RCT), Hydroxyurea Adherence for Personal Best in Sickle Cell Disease “HABIT”, is designed to test the efficacy and sustainability of a CHW intervention augmented by tailored text messages to improve HU adherence (primary outcome), youth HRQOL, self-management responsibility concordance, acute hospital use and self-reported disease symptoms (secondary outcomes). We hypothesize that, compared to the control group receiving standard care plus education handouts on SCD and on HU, at 6 months dyads randomized to the HABIT intervention will demonstrate (1) improved HU adherence measured by progress of HbF to their individualized Personal Best target level and increased proportion of days covered by HU (pharmacy records) (primary outcome); (2) sustained improvement over the subsequent 6 month period; (3) improved generic and disease-specific HRQOL and greater self-management responsibility dyad concordance; and (4) improved health status measured by decreased total length of stay for acute hospitalizations and emergency room encounters and decreased self-reported fatigue, pain interference and pain intensity that is sustained at 12 months (all secondary outcomes). Using focus group and individual interview qualitative methods, we will examine the perspectives of youth, parents and CHWs regarding the impact and sustainability of developing a HU habit (exploratory aim).

### Theoretical framework

Two theoretical frameworks guide the research. The Self and Family Management Framework [70, 71] guides the study design for the randomized controlled trial. Designed to better understand and improve self- and family self-management of chronic conditions, the framework addresses key risk factors for youth and family such as social and psychosocial stressors and incomplete knowledge of drug benefit. Recently updated, the revised framework [71] highlights processes such as activating community resources (e.g., CHWs) to promote self-management of chronic illness and differentiates proximal (e.g., HU adherence) from distal (e.g., HRQOL, health status) outcomes.

In this study we will examine the efficacy and sustainability of a community intervention delivered by CHWs (process) on proximal (adherence behaviors) and distal (HRQOL, self-management responsibility concordance and health status) outcomes. Youth ages 10-18 years are targeted for the HABIT intervention because transition of self-management responsibility occurs throughout this time [20, 42, 47]. Habit formation, a process by which a behavior becomes automatic through ongoing repetition, is based on three components: a cue, the behavior itself, and the inherent reward of regular performance of the behavior [72]. Integration of the CHW and text-messaging components of the HABIT intervention addresses cue and behavior. We will use dyad self-report measures at bi-monthly study clinic visits and qualitative assessment at months 6 and 12 to better understand inherent reward.

The PRECEDE-PROCEED model of health program planning and evaluation [73] is a widely used framework in public health when studying behavior change. The PRECEDE portion (examination of the predisposing, enabling and reinforcing factors) guides the qualitative interviews and analysis for the exploratory aim. This model has been widely used in evaluation of health interventions such as physical activity [74] and obesity prevention [75, 76]. In the HABIT study, we use PRECEDE to gain better understanding of the effect of the intervention and its sustainability from the perspectives of the dyads who received the intervention and the CHWs who delivered it. These perspectives will provide context for interpretation of the study’s quantitative findings and, should the HABIT intervention be efficacious, its potential for broader dissemination.

### Study participants

In total, 104 parent youth dyads will participate in this study. Both dyad members must meet all inclusion and exclusion criteria. Dyads are eligible for participation if the youth is (1) between the ages of 10 and 18 years and (2) diagnosed with SCD type HbSS or HbS-B^0^ thalassemia, (3) has been prescribed HU for a minimum of 18 months, (4) current HU dose (mg/kg/day) is within 5% of dose at Personal Best HbF, and has been stable for the preceding 3 months, (5) pre-enrollment HbF is at least 15% below the Personal Best value based on the calculated average of 2 HbF assessments over the preceding 12 months, (6) is able to use a cell phone with text message capability, (7) can speak and read either English or Spanish, and (8) is willing to participate in clinic and CHW study visits. Parents are eligible for the study if their youth meets all inclusion criteria and if the parent (8) speaks either English or Spanish, (10) is willing to participate in clinic and CHW study visits, and (11) the family expects to reside in their present community for the next one and a half years. The final qualifying inclusion criterion (12) at the Month 0 study visit is a HbF at least 15% below the youth’s Personal Best value.

Dyads are excluded from study participation if the youth’s (1) age is less than 10 years or greater than 18 years, (2) not prescribed HU, (3) has had less than 2 assessments of HbF level over the past year, (4) has had a blood transfusion within 3 months preceding enrollment, (5) if the youth does not currently reside with the parent or legal guardian, (6) is a sibling of a youth enrolled in the study, or (7) has cognitive impairment of greater than two grade levels below what is expected by age. Female youth are also excluded if they are (8) sexually active and not using a form of contraception due to HU’s teratogenic risk to the fetus or are (9) pregnant. Parents are excluded from study participation if (10) he/she is not the primary caregiver or if the youth is in foster care.

### Recruitment

Prior to recruitment the study received approval from the Institutional Review Boards at each study site. Youth/parent dyads will be recruited from four pediatric SCD Centers: Columbia University Irving Medical Center (CUIMC), NY, NY; Montefiore Hospital, Bronx, NY; Cohen Children’s-Northwell Health, Queens, NY; and Children’s Hospital of Philadelphia, Philadelphia, PA. Considerable diversity in patient population exists within and among sites, including African American, African, West Indian and Latino families. The patient volume at each center varies; however, each site has a sufficient number of youth who will both meet eligibility criteria and be interested in study participation. Each site has wireless Internet at their clinic to allow subject use of iPad technology for direct entry of survey data into a Research Electronic Data Capture (REDCap) database designed for HABIT. Study visits will take place in the SCD outpatient clinic setting.

Clinic rosters of youth between the ages of 10-18 years on HU therapy will be assessed for patient eligibility. Parents of youth passing the initial screening will be telephoned for invitation to study participation. Of those interested, full eligibility criteria will be confirmed at their clinic visit. Numbers and reasons for declining the offer to participate will be tracked by site and cumulatively. Prior to study enrollment, parent/legal guardian consent and youth assent will be obtained by a research team member at each site for study participation. Consent and assent forms will be available in either English or Spanish, based on subject preference.

### Randomization process

A 1:1 randomization plan was performed before the start of the trial using a computer-generated assignment sequence in permuted blocks of eight [77] stratified by study site. The randomization plan will be maintained centrally at the Columbia site. Following subject enrollment and confirmation that the youth’s HbF meets the Month 0 qualifying study criterion, site coordinators will contact the Columbia PI for dyad assignment and study ID number. For subjects assigned to receive the intervention, site coordinators will contact the subject and CHW to facilitate prompt scheduling of the first CHW visit. Youth-parent dyads, study coordinators and site PIs cannot be blinded to group assignment, as CHWs are supervised by the study coordinators and serve as a bridge to the clinical staff. While it is not possible to blind dyads to group assignment due to the nature of the HABIT intervention which includes visits with CHWs, dyads will be blinded to study hypotheses. All involved in data analysis will be blinded to dyad assignment.

### Data collection

**Fig. 1:**
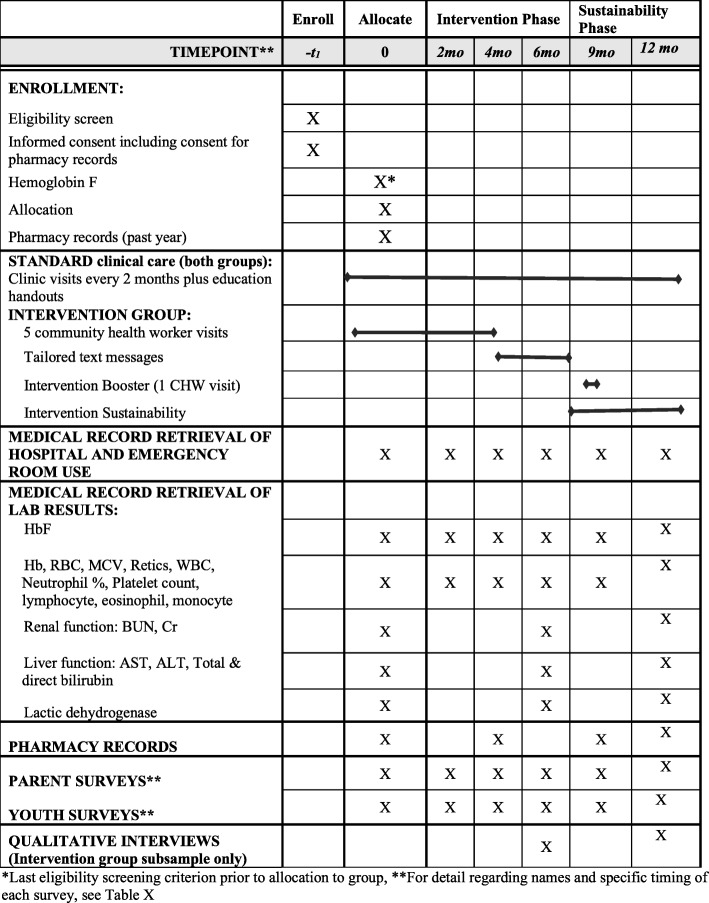
HABIT Efficacy trial

Figure 1 provides detail regarding the schedule of HABIT enrollment, interventions, and ongoing assessments obtained by parent and youth self-reported surveys. Prior to study initiation a Research Electronic Data Capture (REDCap) database was built to accommodate subject eligibility screening for the HABIT study and entry of study visit data for enrolled subjects by dyad member and study coordinator. Access to the password-protected database is site- and user-specific to assure protection of personal health information. Data sources include parent and youth self-reported survey responses directly entered by iPad into the REDCap database, laboratory data and emergency room, hospital admission and blood transfusion utilization extracted from the electronic medical record, HU prescription refill information obtained from subjects’ local pharmacies and individual interviews with a purposive sample of parent youth dyads assigned to the HABIT intervention at 6 months and 12 months. Central access to study data excludes subject personal identifiers.

#### Survey data

Table 1 lists each survey, indended respondent, and timepoints for completion over the duration of the study. Some surveys (e.g., pain interference) will be completed at every study visit from 0 to 12 months, whereas other surveys (e.g., HRQOL) will be completed at months 0, 4, 9 and 12. Each is available in Spanish or English. Measures unavailable in Spanish (all but the PedsQL) were translated to Spanish, piloted as part of the HABIT feasibility trial, and have been previously described [69]. For the efficacy trial, four additional pediatric measures from the Patient Reported Outcomes Measurement Information System (PROMIS) will be used to assess pain interference, pain intensity, fatigue and depressive symptoms and one adult PROMIS measure, emotional distress/depression, were added to the battery of surveys. A brief description of each PROMIS survey follows:

- Pain Interference – Short Form: eight item, 5-point Likert scale developed as part of the PROMIS initiative; *T*-score scale with mean = 50 and standard deviation = 10 [78] for youth and parent proxy versions, responsive to changes in SCD health status [79–81].
- Pain Intensity: one item visual analogue scale regarding perception of pain intensity over the past 7 days with scores ranging from 1 to 10. The scale was developed as part of the PROMIS initiative.
- Fatigue-Short Form: 10 item, 5-point Likert scale developed as part of the PROMIS initiative; *T*-score scale with mean = 50 and standard deviation = 10 [82] for youth and parent proxy versions.
- Depressive symptoms – Short Form (Youth completion only): eight item 5 point Likert scale developed as part of the PROMIS pediatrics project [82]; *T*-score scale with mean = 50 and standard deviation = 10.
- Emotional distress/depression – Short Form (Parent completion only): four item, 5-point Likert scale developed as part of the PROMIS initiative; *T*-score scale with mean = 50 and standard deviation = 10.

**Table 1:** Survey completion by month for parents and youth

Survey Name	Respondent		Study visit month					
	Parent	Youth	0	2	4	6	9	12
Demographic survey	•		•					
Resource use questionnaire	•		•	•	•	•	•	•
Medication barriers scale	•	•	•			•		•
Sickle cell family responsibility	•	•	•		•		•	•
PedsQL generic core scales^a^	•	•	•		•		•	•
PedsQL sickle cell disease module^a^	•	•	•		•		•	•
Pain interference	•	•	•	•	•	•	•	•
Pain intensity	•	•	•	•	•	•	•	•
Fatigue	•	•	•	•	•	•	•	•
Depressive symptoms		•	•	•	•	•	•	•
Emotional distress	•		•	•	•	•	•	•
Evaluation^b^	•	•						•
Comment cards^c^	•	•		•	•	•	•	•

All surveys available in Spanish and English; ^a^Different survey versions depending on youth age (8-12 years; 13-18 years); ^b^Different survey versions for intervention and control groups; ^c^Completed by intervention group only

#### Medical record data extraction

Following each study visit, laboratory data will be extracted from the electronic medical record as detailed in Fig. 1: Months 0, 2, 4, 6, 9, 12: HbF (%), hemoglobin (%), red blood cell count, mean corpuscular volume, reticulocyte count, white blood cell count, neutrophils (%), platelet count, lymphocyte (%), eosinophils (%), and monocyte (%); Months 0, 6, 12: in addition to the laboratory studies detailed above, blood urea nitrogen, creatinine, aspartate aminotransferase (AST), alanine aminotransferase (ALT), total and direct bilirubin and lactic dehydrogenase (LDH) will be assessed. HbF will be excluded from assessment at a study visit if the subject had received a blood transfusion within 90 days of the study visit or had an acute pain episode within the past 2 weeks. In addition, records of hospitalizations and/or emergency department use will be extracted from the medical record.

#### Prescription refill data

At each study visit, study coordinators will inquire if the subject’s pharmacy has changed and, if so, the parent will be asked to update their consent for release of pharmacy prescription refill information. Pharmacies will be contacted at study entry for the prior year’s prescription information and at months 3, 6, 9 and 12 for prescription information concurrent with the study period.

#### Qualitative interviews

After completing the 6-month study visit and at study completion (12 months), a purposive sample of parent-youth dyads from each clinical site will participate in individual interviews (a total of 10 dyads reflecting the 4 clinical sites at each timepoint). Using uniform interview guides, interviews will be conducted by the coordinator at each site either face to face at the clinic or by telephone to gain dyad perspective regarding the inherent rewards of HU adherence. Interviews for parents and youth will be conducted separately and each is expected to last approximately 20-30 min. Subjects will be compensated for their time. The interview guide for interviews at month 6 will be directed to intervention impact. The interview guide at month 12 interviews will be geared toward the sustainability of the habit. In addition, we will conduct two focus groups by webinar with all study CHWs across sites at two time points: study mid-point (18 months) and end-point (36 months). The focus group interview guide questions will solicit CHW experiences with the dyads regarding the predisposing, enabling and reinforcing factors and their relationship to intervention impact and sustainability.

### Clinical care visits

Following randomization, all dyads will receive routine clinic-based care and monitoring of HbF levels at months 2, 4, 6, 9 and 12. The intervention and control groups will receive the same educational materials about SCD and HU and complete the same study questionnaires and laboratory assessments. In addition, the intervention group will receive five visits from CHWs during the first 4 months of the study as described below, as well as a booster CHW visit at month 9. Following completion of the initial CHW visits and identification of an existing daily habit on which HU adherence can be built, dyads will receive daily text messages to support development of the HU habit.

### HABIT intervention

**Fig. 2:**
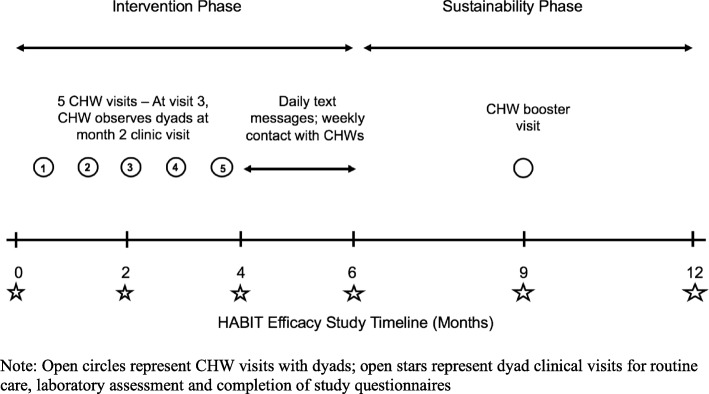
Timeline of the HABIT Intervention

Dyads assigned to the intervention group will receive a multi-component intervention based on CHW visits and support, initiation of a habit to foster HU adherence, and daily text messages to reinforce the habit. While each visit is focused, it is also tailored based on the needs of the dyad. Figure 2 provides detail regarding the HABIT intervention. The intervention occurs over the first 6 months of the study; months 7 to 12 are observational to examine sustainability of HbF improvement over time. Following randomization, dyads assigned to the intervention group will be contacted by the study coordinator at each site to schedule a time and location for the first CHW visit. While most visits will occur at the dyad’s home [54], location of visits will be based on dyad preference. Alternatively, CHW visits may also occur in a local community-based organization, coffee shop or in a quiet site within their clinical space. CHW visits 1, 2, 4 and 5 are guided by a checklist of tasks to be completed during the visit. Visits 1 and 2, accomplished within the first 2 months of study entry, include establishing the dyad/CHW relationship, assessment of family structure, sources of social support, and need for referrals (e.g., housing, food insecurity, mental health), review HU and SCD educational handouts received at study enrollment with the dyad, and assess barriers to HU. The CHWs will also introduce the concept of reaching Personal Best HbF through improved adherence. At the 2-month clinical study visit, CHWs will meet the dyad at the clinic to observe their communication and relationship with their healthcare provider (visit 3).

Visits 4 and 5 focus on delegation of self-management responsibility for HU, youth-parent partnership for self-management, and identification of a habit on which HU adherence can be built. The identified habit is trialed between visits 4 and 5. If deemed feasible, at visit 5 text messages tailored by language (Spanish or English), content (set by the subject) and time of delivery (specfic to the habit) are developed. Following conclusion of visit 5, daily text messages to parent and youth will be initiated using an automated text message system (OnSolve LLC, Secausus, NJ). During the texting phase, parents and youth will each receive a separate weekly automated text asking about the number of days (0 to 7) the youth has taken their HU. Dyad response is recorded within the OnSolve system. Based on parent and youth responses to the weekly text, the CHW will provide feedback regarding their adherence. If adherence is poor, if the dyad fails to respond to the weekly text or the dyad reports disparate answers, the CHW will call the dyad to discuss the circumstances that led to adherence problems and help the dyad to problem solve to improve adherence. If dyad response to the weekly text denotes good daily adherence, the CHW will provide motivational feedback to the dyad to maintain their habit. Following the 6 month clinic visit, the text message component of the intervention will conclude.

A CHW booster visit will occur at 9 months. The CHW will visit the dyad either at home or other preferred location to assess the dyad’s success in continuing the HU habit following discontinuation of the intervention, need for and utilization of social support. The CHW will also review HbF values with the dyad and overall progress to Personal Best HbF.

### CHW training and supervision

A study orientation meeting of all site PIs, coordinators and CHWs will be held prior to subject enrollment. At that meeting, study principal investigators will present the study’s main goals and strategies, including lessons learned from the HABIT feasibility trial [53, 54] review the study protocol and address issues at any of the sites.

#### CHW training

Following hiring, CHWs from all sites will participate in a 4-day training session. Training will use the structured training curriculum from the HABIT feasibility study [69]. The first 2 days will serve as a “refresher” regarding CHW core concepts such as CHW role and responsibility, engagement with families, and role boundaries. Days 3-4 of training will provide project-specific training, to include HABIT study goals, rationale and approach, schedule and goal of each CHW visit, information about SCD and HU. Project specific skills (e.g., establishing relationships with dyads, helping dyads to work together to address barriers to HU and identify established daily patterns on which to build a HU habit, communication with the medical team) will be addressed through discussion and role playing. Developmentally appropriate self-management expectations for youth at varying age levels will be presented. The added benefit of group training is for the CHWs to form a group allied for sharing experiences during supervision sessions over the course of the project. For any “red flag” issues (e.g., truancy, depression, drug use, potential violence), CHWs will be directed to immediately inform their site coordinator for referral to the clinic’s social services.

#### CHW supervision

CHWs will receive supervision both at the local study site level and at the overall project level. Each site PI will meet with their respective CHWs a minimum of once per month; for the remaining weeks, site coordinators will meet with CHWs weekly for caseload updates, communicate with CHWs regarding subject HbF progress, information that may trigger clinical or social worker contact (e.g., turmoil in the home, missed school due to recurring disease symptoms or truancy), and to review all CHW visit forms and automated text messaging reports. In addition, the principal investigators at the Columbia site will lead monthly CHW group supervision sessions delivered via webinar for all CHWs to reinforce training components and ongoing case supervision. CHWs will share their experiences in delivering the intervention to enable all CHWs to learn from the collective experiences.

### Intervention fidelity

Intervention fidelity will be assured through a structured intervention protocol, ongoing CHW supervision by site principal investigators (PIs) and study coordinators, and monthly communication with program PIs. The HABIT operations manual contains CHW encounter forms to structure and document the content of each scheduled encounter, monthly schedules and objectives to guide family discussion, key messages to promote HU adherence and youth-parent self-management partnership, exemplars of cues for text messaging reminders, and information for families on SCD and HU to be reviewed with dyads. CHW delivery of intervention and completed visit forms will be reviewed weekly during supervision. Feedback and guidance will be provided to CHWs for each dyad. Monthly conference calls will be held for coordinators and for site PIs to oversee enrollment schedules, scheduling and logistics of home visits, obtaining pharmacy refill data, study procedures and to problem-solve, as needed. To assure validity of HbF values, the subject’s transfusion history will be reviewed during each coordinator call. Levels will be discarded if the subject had been transfused within the preceding 90 days, per standard HU protocols [55].

### Outcome measures

Medication adherence, the primary outcome, will be measured as distance from Personal Best HbF (biomarker) and proportion of days when HU was available (pharmacy records). Secondary outcomes will be parent and youth self-report of generic (PedsQL [83]) and disease-specific (PedsQL sickle cell disease module [84]) HRQOL, greater self-management responsibility concordance (Sickle Cell Family Responsibility, a measure based on the Diabetes Family Responsibility Questionnaire [44, 85]) and improved health status. Improved health status will be measured by electronic medical record documentation of SCD related acute hospitalizations and emergency room use and parent and youth self-report of youth fatigue, pain interference and pain intensity.

### Sample size and statistical power

Based on findings from the HABIT feasibility trial [53, 54], we estimated the statistical power to compare score changes from Month 0 to Months 6, and from Months 6 to 12 to test both efficacy and sustainability between the intervention and control groups using a linear mixed model. All power analyses were based on a 2-sided test and α < 0.05. We assumed that each outcome measure was highly correlated (*r* = 0.7) at different times and the clustering with each site was low with an intra-cluster coefficient (ICC) of 0.1. We also assumed that this study will have 4 sites and each site will meet its enrollment target. Stratified by study site, subjects will be randomly assigned 1:1 to either the intervention or control group with 20% attrition rate by the 12th month. For the difference in differences (DID) intervention efficacy analysis, there is 83.2% power to detect a medium effect size of 0.6. Statistical power was also estimated to compare the trend of score changes between the intervention and control groups from Month 0 to Month 12 for outcomes that will be measured every 2 months. For the DID analyses for intervention sustainability, there will be 85.6% power to detect a small effect size of 0.25.

### Data management and analysis

Subjects will be assigned a unique identification number for direct entry of survey data via iPad to a REDCap database (http://project-redcap.org/), a web-based research application which is supported at Columbia, and stored on a CUIMC Institutional Review Board server. Clinical data obtained during study visits will be entered into a REDCap study database by study coordinators at each site. Each study site will have full access to site specific data with the lead site (Columbia) having access to de-identified data from all study sites. Data analyses will be performed using SAS version 9.4 (SAS Institute, Cary, NC).

All quantitative analyses are based on intention-to-treat with all randomized subjects included in the analyses. Following downloading of data from REDCap and merging of de-identified data across study sites, descriptive statistics will be used to profile outcome measures at each data collection point for the intervention and control groups. Distributions of all outcome variables will be made at the observational level instead of at the subject level. Total hospital length of stay, hospitalizations and emergency room visits will be compared during three time periods: months 0-12 of the year prior, months 0-6 and 7-12. The main analysis will be difference-in-difference (DID), comparison of changes in outcomes at month 6 from month 0 (improvement) and month 12 from month 6 (sustainability) between the intervention and the control group. We will use a linear mixed model or generalized linear mixed model for data analysis. Linear mixed models are used for most continuous outcomes (e.g., HRQOL score) and generalized linear mixed models are for categorical outcomes (e.g., whether or not a subject had an urgent outpatient visit with logit link function) or for outcomes with skewed distribution (e.g., length of stay with log link function). A site-specific random effect will be incorporated into the mixed models to control for clustering within each site. The mixed models are also used for outcomes that will be assessed every 2 months from 0 to 12 month visit to examine the difference in trend during the follow-up period. The mixed model is used to address the hierarchical data structure of multiple observations for each subject, multiple subjects in each family (i.e., youth-parent dyads), as well as for repeated measures data. Other variables will be explored, e.g. age, gender and family structure, to identify those that serve as mediators or moderators of the intervention’s effect.

Subject attrition or other missing data (e.g., survey or invalid HbF result due to recent transfusion) will be addressed by a plan to: (1) apply a mixed model to include all subjects in the analysis; (2) conduct a sensitivity analysis to estimate magnitude and direction of bias by imputing missing outcomes; and (3) ask dyads who do not complete follow-up interviews about their reasons why and include such information in the model to correct the bias.

For qualitative data obtained from focus groups and individual interviews, data will be analyzed using content analysis with codes independently created based on line by line analysis, checked for inter-rater agreement, and resolved through consensus [86, 87]. Codes will be sorted into categories by PRECEDE constructs: predisposing, enabling, and reinforcing, and by themes identified [73] to allow better understanding of the impact and sustainability potential of the HABIT intervention. The research team will ensure the credibility, confirmability, dependability and transferability of the qualitative findings. To assure credibility we will conduct peer debriefing and triangulate findings across data sources (focus group, individual interview, survey data), use member checking and sharing of data interpretation with participants for accuracy. Triangulation of findings will enhance confirmability of findings. Data will be analyzed concurrently with the interview process, thereby using a constant comparative approach. An audit trail and extensive field notes will be maintained to facilitate transferability of findings. All transcripts and field notes will be analyzed using NVivo™ (QSR International, Victoria, Australia) software.

### Safety considerations

The study poses minimal risk to subjects. However, it is possible that during dyad home visits, CHWs will discover “red flag” issues such as truancy, suicidal ideation, illicit drug use, or potential violence. CHWs will be directed to immediately report these concerns to their study site coordinator for referral to the clinic’s social services.

### Monitoring

A five member Data Safety and Monitoring committee (2 nurse scientists, 1 biostatistician, 1 pediatric sickle cell disease specialist, 1 pediatrician) will meet quarterly throughout the trial to monitor for evidence of possible harm to subjects, track participant accrual rates, and to monitor the primary and secondary outcomes for early evidence of efficacy, harm or futility. To accomplish this, summaries of data quality, accrual, adherence, distribution of baseline factors, harms, study endpoints and other analyses as requested will be prepared for review by the Data Safety Monitoring Committee. As a minimal risk study that been piloted for feasibility at 2 study sites, adverse consequences such as drug toxicity of a clinically prescribed medication or other medical consequences are highly unlikely. All laboratory data will be reviewed and interpreted by physicians or advanced practice nurses on our study team who will make decisions regarding the need for subject follow up. Should an adverse event occur, the event will be reported according to the requirements of the Columbia University Medical Center Institutional Review Board and the Institutional Review Boards of the collaborating study sites. In addition, the Data Safety and Monitoring Committee will be notified of the adverse event and all adverse events and their resolution reviewed at its next quarterly meeting.

The primary outcome of the trial, improved hydroxyurea adherence at 6 months as measured by improved personal best fetal hemoglobin (HbF), is the basis for the formal interim analysis plan that follows. Two interim analyses and one final analysis are planned and will be performed when 0.35, 0.65 and 1.0 fraction of the total number of participants will have finished the 6-month assessment of the primary outcome. Based on a targeted sample of 104 dyads, analyses will be conducted upon 6 month completion of 35, 70 and 104 subjects respectively.

Based on these findings as well as other factors such as intervention effect on secondary outcome measures and development of any new external scientific evidence with regard to hydroxyurea adherence for youth with sickle cell disease, the Data Safety and Monitoring committee will determine whether to allow the study to continue.

### Ethics approval

The IRBs at each of the four study sites have approved the study protocol and deemed that it poses no excess study risk to subjects. Eligible parent youth dyads willing to participate will sign a written consent and/or assent. All subjects will be informed that their participation is voluntary, that they may withdraw from the study at any time, and that their survey responses will not influence their usual medical care.

### Trial status

The HABIT study is currently ongoing. Recruitment commenced in September 2018 and is planned to continue for a 24 month period. Trial results will be disseminated by publications in relevant peer-review interdisciplinary journals and by presentations at regional and national conferences.

## Discussion

To date, HU remains the primary disease modifying therapy for youth with SCD with proven efficacy. In 2014 offering HU to youth with SCD became standard recommended practice [7]. Since that time, HU has been increasingly prescribed for youth with SCD [88]. For HU to achieve its therapeutic potential for youth with SCD, adherence must be optimized. Using 2005-2012 Medicaid data from six states, findings of a recent study suggest that less than 20% of youth for whom HU was prescribed received at least 300 days of medication [89]. Barriers to HU are pervasive and may differ for parents and youth [28]. This points to the critical need for interventions to improve adherence for these vulnerable youth. Dyad engagement with chronic disease self-management improves youth health outcomes by not only improving adherence to prescribed medications but also by building capacity for problem solving and fostering resilience when challenges occur [39]. If efficacious, the HABIT intervention has the potential to improve the lives of youth with SCD.

The HABIT efficacy trial builds upon a feasibility trial that study subjects deemed feasible and acceptable. Extension of the trial to four study sites increases generalizability of study findings and potential for broader implementation in real world settings. However, the proposed trial has several limitations. Neither study investigators nor subjects are blinded. As CHWs have a major role in the intervention, blinding is not possible. Our procedure for allocation concealment minimizes potential bias regarding group assignment. Blinding will be maintained during the data analysis process. The youth exclusion criterion for cognitive impairment is defined as greater than two grade levels below what is expected by age rather than by formal cognitive testing. While the sample size is sufficient to test efficacy of the multi-component intervention, it is not powered to compare the effect of individual intervention components of CHW support and tailored text message reminders. Qualitative focus group data at two time points post intervention will provide information about any additive impact from text messaging. A peer support group for parents and youth is not provided as part of the study protocol. Attending a peer support group would likely be difficult for this multi-ethnic sample, on top of busy families and working parents. CHW coaching youth and parent to identify and develop individual support is intended to establish longer-term social support. If the intervention is efficacious but not sustainable at 12 months, extended CHW support would require further testing.

## Data Availability

Not applicable.

## Abbreviations

ALT: Alanine aminotransferase
AST: Aspartate aminotransferase
CHW: Community health worker
HABIT: Hydroxyurea Adherence for Personal Best in Sickle Cell Disease
HbF: Fetal hemoglobin (Hemoglobin F)
HRQOL: Health related quality of life
HU: Hydroxyurea
LDH: Lactic dehydrogenase
PROMIS: Patient Reported Outcomes Measurement Information System
REDCap: Research Electronic Data Capture
SCD: Sickle cell disease

## References

[1] Hassell KL (2010). Population estimates of sickle cell disease in the U.S. Am J Prev Med.

[2] Brousseau DC (2010). The number of people with sickle-cell disease in the United States: national and state estimates. Am J Hematol.

[3] Brousseau DC (2010). Acute care utilization and rehospitalizations for sickle cell disease. JAMA.

[4] Raphael JL (2012). Shorter hospitalization trends among children with sickle cell disease. Pediatr Blood Cancer.

[5] Lanzkron S, Carroll CP, Haywood C (2013). Mortality rates and age at death from sickle cell disease: U.S., 1979-2005. Public Health Rep.

[6] Hamideh D, Alvarez O (2013). Sickle cell disease related mortality in the United States (1999-2009). Pediatr Blood Cancer.

[7] Yawn BP (2014). Management of sickle cell disease: summary of the 2014 evidence-based report by expert panel members. JAMA.

[8] Thornburg CD, Calatroni A, Panepinto JA (2011). Differences in health-related quality of life in children with sickle cell disease receiving hydroxyurea. J Pediatr Hematol Oncol.

[9] McGann PT, Ware RE (2011). Hydroxyurea for sickle cell anemia: what have we learned and what questions still remain?. Curr Opin Hematol.

[10] Ware RE (2010). How I use hydroxyurea to treat young patients with sickle cell anemia. Blood.

[11] Candrilli SD (2011). Hydroxyurea adherence and associated outcomes among Medicaid enrollees with sickle cell disease. Am J Hematol.

[12] Brandow AM, Panepinto JA (2011). Monitoring toxicity, impact, and adherence of hydroxyurea in children with sickle cell disease. Am J Hematol.

[13] Ritho J (2011). Hydroxyurea use in patients with sickle cell disease in a Medicaid population. Am J Hematol.

[14] Thornburg CD (2010). Adherence to hydroxyurea therapy in children with sickle cell anemia. J Pediatr.

[15] Walsh KE (2014). Medication adherence among pediatric patients with sickle cell disease: a systematic review. Pediatrics.

[16] Green NS (2016). Decreased fetal hemoglobin over time among youth with sickle cell disease on hydroxyurea is associated with higher urgent hospital use. Pediatr Blood Cancer.

[17] Bogart LM (2010). Conspiracy beliefs about HIV are related to antiretroviral treatment nonadherence among african american men with HIV. J Acquir Immune Defic Syndr.

[18] Simons LE, Blount RL (2007). Identifying barriers to medication adherence in adolescent transplant recipients. J Pediatr Psychol.

[19] Oyeku SO (2013). Parental and other factors associated with hydroxyurea use for pediatric sickle cell disease. Pediatr Blood Cancer.

[20] McQuaid EL (2003). Medication adherence in pediatric asthma: reasoning, responsibility, and behavior. J Pediatr Psychol.

[21] Delgado EM (2014). Parental asthma education and risks for nonadherence to pediatric asthma treatments. Pediatr Emerg Care.

[22] Wisk LE, Witt WP (2012). Predictors of delayed or forgone needed health care for families with children. Pediatrics.

[23] Reach G (2005). Role of habit in adherence to medical treatment. Diabet Med.

[24] McGrady ME, Hommel KA (2013). Medication adherence and health care utilization in pediatric chronic illness: a systematic review. Pediatrics.

[25] Desai M, Oppenheimer JJ (2011). Medication adherence in the asthmatic child and adolescent. Curr Allergy Asthma Rep.

[26] Taddeo D, Egedy M, Frappier JY (2008). Adherence to treatment in adolescents. Paediatr Child Health.

[27] Quittner AL (2014). Pulmonary medication adherence and health-care use in cystic fibrosis. Chest.

[28] Smaldone A, Manwani D, Green NS (2019). Greater number of perceived barriers to hydroxyurea associated with poorer health-related quality of life in youth with sickle cell disease. Pediatr Blood Cancer.

[29] Rothman RL (2008). Self-management behaviors, racial disparities, and glycemic control among adolescents with type 2 diabetes. Pediatrics.

[30] McQuaid EL, Landier W (2018). Cultural issues in medication adherence: disparities and directions. J Gen Intern Med.

[31] Iuga AO, McGuire MJ (2014). Adherence and health care costs. Risk Manag Healthc Policy.

[32] Pai AL, Drotar D (2010). Treatment adherence impact: the systematic assessment and quantification of the impact of treatment adherence on pediatric medical and psychological outcomes. J Pediatr Psychol.

[33] Anders DG (2016). Hydroxyurea use in young children with sickle cell anemia in New York state. Am J Prev Med.

[34] Estepp JH (2017). A clinically meaningful fetal hemoglobin threshold for children with sickle cell anemia during hydroxyurea therapy. Am J Hematol.

[35] Estepp JH (2014). Improved hydroxyurea effect with the use of text messaging in children with sickle cell anemia. Pediatr Blood Cancer.

[36] Creary SE (2014). A pilot study of electronic directly observed therapy to improve hydroxyurea adherence in pediatric patients with sickle-cell disease. Pediatr Blood Cancer.

[37] Inoue S (2016). Adherence to hydroxyurea medication by children with sickle cell disease (SCD) using an electronic device: a feasibility study. Int J Hematol.

[38] Pernell BM (2017). Improving medication adherence with two-way short message service reminders in sickle cell disease and asthma. A feasibility randomized controlled trial. Appl Clin Inform.

[39] Lozano P, Houtrow A (2018). Supporting self-management in children and adolescents with complex chronic conditions. Pediatrics.

[40] Naar-King S (2009). Allocation of family responsibility for illness management in pediatric HIV. J Pediatr Psychol.

[41] Anderson B (1997). Parental involvement in diabetes management tasks: relationships to blood glucose monitoring adherence and metabolic control in young adolescents with insulin-dependent diabetes mellitus. J Pediatr.

[42] Orrell-Valente JK (2008). At what age do children start taking daily asthma medicines on their own?. Pediatrics.

[43] Walders N, Drotar D, Kercsmar C (2000). The allocation of family responsibility for asthma management tasks in African-American adolescents. J Asthma.

[44] Anderson BJ (1990). Assessing family sharing of diabetes responsibilities. J Pediatr Psychol.

[45] Anderson BJ (1999). An office-based intervention to maintain parent-adolescent teamwork in diabetes management. Impact on parent involvement, family conflict, and subsequent glycemic control. Diabetes Care.

[46] Treadwell MJ (2005). Barriers to adherence of deferoxamine usage in sickle cell disease. Pediatr Blood Cancer.

[47] Markowitz JT, Garvey KC, Laffel LM (2015). Developmental changes in the roles of patients and families in type 1 diabetes management. Curr Diabetes Rev.

[48] Hanghoj S, Boisen KA (2014). Self-reported barriers to medication adherence among chronically ill adolescents: a systematic review. J Adolesc Health.

[49] Zimmerman SA (2004). Sustained long-term hematologic efficacy of hydroxyurea at maximum tolerated dose in children with sickle cell disease. Blood.

[50] Hankins JS (2014). From infancy to adolescence: fifteen years of continuous treatment with hydroxyurea in sickle cell anemia. Medicine (Baltimore).

[51] Ferster A (2001). Five years of experience with hydroxyurea in children and young adults with sickle cell disease. Blood.

[52] Platt OS (2008). Hydroxyurea for the treatment of sickle cell anemia. N Engl J Med.

[53] Smaldone A (2018). HABIT, a randomized feasibility trial to increase hydroxyurea adherence, suggests improved health-related quality of life in youths with sickle cell disease. J Pediatr.

[54] Green Nancy S., Manwani Deepa, Matos Sergio, Hicks April, Soto Luisa, Castillo Yina, Ireland Karen, Stennett Yvonne, Findley Sally, Jia Haomiao, Smaldone Arlene (2017). Randomized feasibility trial to improve hydroxyurea adherence in youth ages 10-18 years through community health workers: The HABIT study. Pediatric Blood & Cancer.

[55] Ware RE (2002). Predictors of fetal hemoglobin response in children with sickle cell anemia receiving hydroxyurea therapy. Blood.

[56] Peretz PJ (2012). Community health workers as drivers of a successful community-based disease management initiative. Am J Public Health.

[57] Findley SE (2012). Building a consensus on community health workers’ scope of practice: lessons from New York. Am J Public Health.

[58] Palmas W (2015). Community health worker interventions to improve glycemic control in people with diabetes: a systematic review and meta-analysis. J Gen Intern Med.

[59] Verhagen I (2014). Community health worker interventions to improve access to health care services for older adults from ethnic minorities: a systematic review. BMC Health Serv Res.

[60] Kim K (2016). Effects of community-based health worker interventions to improve chronic disease management and care among vulnerable populations: a systematic review. Am J Public Health.

[61] Raphael JL (2013). The role of lay health workers in pediatric chronic disease: a systematic review. Acad Pediatr.

[62] Hsu LL (2016). Community health workers as support for sickle cell care. Am J Prev Med.

[63] Poorman E (2015). Use of text messaging for maternal and infant health: a systematic review of the literature. Matern Child Health J.

[64] Badawy SM (2017). Text messaging and mobile phone apps as interventions to improve adherence in adolescents with chronic health conditions: a systematic review. JMIR Mhealth Uhealth.

[65] Badawy SM, Kuhns LM (2017). Texting and mobile phone app interventions for improving adherence to preventive behavior in adolescents: a systematic review. JMIR Mhealth Uhealth.

[66] Smaldone A (2015). Adolescent and parent use of new technologies for health communication: a study in an urban latino community. J Public Health Res.

[67] Hood KK (2010). Interventions with adherence-promoting components in pediatric type 1 diabetes: meta-analysis of their impact on glycemic control. Diabetes Care.

[68] Kahana S, Drotar D, Frazier T (2008). Meta-analysis of psychological interventions to promote adherence to treatment in pediatric chronic health conditions. J Pediatr Psychol.

[69] Smaldone A (2016). Study protocol for a randomized controlled trial to assess the feasibility of an open label intervention to improve hydroxyurea adherence in youth with sickle cell disease. Contemp Clin Trials.

[70] Grey M, Knafl K, McCorkle R (2006). A framework for the study of self- and family management of chronic conditions. Nurs Outlook.

[71] Grey M (2015). A revised self- and family management framework. Nurs Outlook.

[72] Gardner B, Lally P, Wardle J (2012). Making health habitual: the psychology of ‘habit-formation’ and general practice. Br J Gen Pract.

[73] Gielen AC, Glanz K, Rimer BK, Viswanath K (2008). Using the PRECEDE/PROCEED model to apply health behavior theories. Health behavior and health education: theory, research and practice.

[74] Slawta JN, DeNeui D (2010). Be a fit kid: nutrition and physical activity for the fourth grade. Health Promot Pract.

[75] Cole RE, Horacek T (2009). Applying precede-proceed to develop an intuitive eating nondieting approach to weight management pilot program. J Nutr Educ Behav.

[76] Manios Y (2012). A systematic approach for the development of a kindergarten-based intervention for the prevention of obesity in preschool age children: the ToyBox-study. Obes Rev.

[77] Broglio K (2018). Randomization in clinical trials: permuted blocks and stratification. JAMA.

[78] Varni JW (2010). PROMIS pediatric pain interference scale: an item response theory analysis of the pediatric pain item bank. J Pain.

[79] Dampier C (2016). Responsiveness of PROMIS(R) pediatric measures to hospitalizations for sickle pain and subsequent recovery. Pediatr Blood Cancer.

[80] Thissen D (2016). Estimating minimally important difference (MID) in PROMIS pediatric measures using the scale-judgment method. Qual Life Res.

[81] Curtis S, Brandow AM (2017). Responsiveness of patient-reported outcome measurement information system (PROMIS) pain domains and disease-specific patient-reported outcome measures in children and adulsts with sickle cell disease. Hematology Am Soc Hematol Educ Program.

[82] Lai JS (2013). Development and psychometric properties of the PROMIS((R)) pediatric fatigue item banks. Qual Life Res.

[83] Varni JW, Seid M, Kurtin PS (2001). PedsQL 4.0: reliability and validity of the pediatric quality of life inventory version 4.0 generic core scales in healthy and patient populations. Med Care.

[84] Panepinto JA (2013). PedsQL sickle cell disease module: feasibility, reliability, and validity. Pediatr Blood Cancer.

[85] Anderson BJ (2009). Dyadic measures of the parent-child relationship during the transition to adolescence and glycemic control in children with type 1 diabetes. Fam Syst Health.

[86] Krippendorff KH (2012). Content analysis: an introduction to its methodology.

[87] Neuendorf KA (2017). The content analysis guidebook.

[88] Brousseau David C., Richardson Troy, Hall Matt, Ellison Angela M., Shah Samir S., Raphael Jean L., Bundy David G., Arnold Staci (2019). Hydroxyurea Use for Sickle Cell Disease Among Medicaid-Enrolled Children. Pediatrics.

[89] Reeves SL (2019). Hydroxyurea use among children with sickle cell anemia. Pediatr Blood Cancer.

